# Targeting Inflammation after Myocardial Infarction: A Therapeutic Opportunity for Extracellular Vesicles?

**DOI:** 10.3390/ijms22157831

**Published:** 2021-07-22

**Authors:** Margarida Viola, Saskia C. A. de Jager, Joost P. G. Sluijter

**Affiliations:** 1 Laboratory of Experimental Cardiology, University Medical Center Utrecht, 3584 CX Utrecht, The Netherlands; a.m.carmoviola@umcutrecht.nl; 2 UMC Utrecht Regenerative Medicine Center, Circulatory Health Laboratory, University Utrecht, 3584 CS Utrecht, The Netherlands

**Keywords:** myocardial infarction, cardiac inflammation, monocyte influx, macrophage polarization, immunomodulatory therapy, extracellular vesicles, mesenchymal stem cell-derived EVs, cardiac progenitor cells derived-EVs

## Abstract

After myocardial infarction (MI), a strong inflammatory response takes place in the heart to remove the dead tissue resulting from ischemic injury. A growing body of evidence suggests that timely resolution of this inflammatory process may aid in the prevention of adverse cardiac remodeling and heart failure post-MI. The present challenge is to find a way to stimulate this process without interfering with the reparative role of the immune system. Extracellular vesicles (EVs) are natural membrane particles that are released by cells and carry different macromolecules, including proteins and non-coding RNAs. In recent years, EVs derived from various stem and progenitor cells have been demonstrated to possess regenerative properties. They can provide cardioprotection via several mechanisms of action, including immunomodulation. In this review, we summarize the role of the innate immune system in post-MI healing. We then discuss the mechanisms by which EVs modulate cardiac inflammation in preclinical models of myocardial injury through regulation of monocyte influx and macrophage function. Finally, we provide suggestions for further optimization of EV-based therapy to improve its potential for the treatment of MI.

## 1. Introduction

Despite decades of research, cardiac repair upon myocardial infarction (MI) injury remains a challenge and ischemic heart disease continues to be one of the leading causes of death worldwide [1]. The immune system has a fundamental role in the post-MI process. Once an ischemic injury occurs, a robust inflammatory cell infiltration is initiated in the heart to remove the dead tissue. This is necessary for the healing of the myocardium to happen, however excessive or persistent inflammation can lead to adverse left ventricle (LV) remodeling and development of heart failure [2].

A growing body of evidence suggests that timely resolution of the inflammatory process may aid in the prevention of adverse cardiac remodeling and heart failure [3]. In fact, circulating inflammatory cytokines, such as interleukin-1 beta (IL-1β), IL-6, and tumor necrosis factor alpha (TNFα), correlate with clinical events in patients with chronic heart failure [4]. Different immunosuppressive drugs, anti-inflammatory, and immunomodulatory interventions have been studied upon MI. Suppressing the immune system has a negative impact on post-MI wound healing processes [5]. However, administration of angiotensin-converting enzyme inhibitors and beta-blockers within the first 24 h following MI is part of the recommended treatment guidelines [6]. Although the primary mechanism of action of these drugs is not directly related to immunomodulation, they have been shown to reduce the circulation of monocytes in preclinical studies, decreasing their infiltration in the ischemic area and, thus, cardiac inflammation [7,8]. Moreover, a recent clinical trial showed that IL-1β inhibitor canakinumab reduced hospitalization for heart failure in patients with previously diagnosed MI [9]. Likewise, clinical trials have shown that the granulocyte colony-stimulating factor (G-CSF), an endogenous cytokine mobilizer of bone marrow granulocytes, improved cardiac function by decreasing scar size and preventing LV remodeling in post-MI patients [10,11]. This confirms the promising potential of anti-inflammatory and immunomodulatory therapies in MI patients during acute and chronic cardiac injury phases. The present challenge is to find a way to stimulate inflammatory resolution after ischemic injury in the heart without interfering with the reparative role of macrophages through the development of more selective immunomodulatory treatments.

Transplantation of stem cells or their derivates into the damaged heart has been studied over the last two decades expecting these cells to regenerate cardiac cells in the ischemic area [12]. Initial animal studies claimed that injected cells could transdifferentiate into cardiomyocytes or other cardiovascular cells [13,14]. On the contrary, later studies showed that the injected cells presented poor engraftment and survival in such an ischemic microenvironment [15] and were rapidly cleared via the venous system [16]. Despite this, cell transplantation presented a modest ability to improve cardiac function, however, the mechanism behind its therapeutic benefit remains unclear [17].

Evidence supported the hypothesis that cell-based therapy may act via paracrine signaling as a conditioned medium derived from these cells increased cardiomyocyte survival in both in vitro co-culture system and in vivo [18,19,20]. Virtually all mammalian cells are able to communicate with each other by secretion of multiple factors, ranging from soluble proteins to extracellular vesicles (EVs). EVs are natural lipid nanoparticles released by cells, which can carry different macromolecules, including proteins and non-coding RNAs. EVs can transport biological cargo, both locally and remotely via the bloodstream, and transfer their content into target cells to modify their behavior [21]. EVs attracted significant attention in recent years as they demonstrated the capacity to mimic the biological effects of their parent cells. Stem and progenitor cell-derived EVs were no exception, and their potential as cell-free therapy started to be investigated for cardiovascular applications [22].

Together with the paracrine hypotheses where EVs play a role, it is thought that the injected cells in the heart can modify cardiac inflammation [23]. According to a recent study, adult stem cell therapy improved heart function due to an acute immune response, linking macrophages to functional improvement [24]. This finding highlights how cell-based therapy can modulate the innate immune system, which prompted the question of whether or not their secreted EVs can act likewise. Interestingly, it was described that endogenous EV release by cardiac cells shaped cardiac inflammation in a mouse model of MI, further demonstrating the potential of EVs in regulating immune response upon MI [25].

In this review, we highlight the role of the innate immune system in post-MI healing, focusing on the function of monocytes and macrophages during cardiac ischemic injury and repair. Next, we summarize and discuss the mechanisms by which the EVs studied as therapy in myocardial repair can modulate monocyte and macrophage function and, thus, cardiac inflammation. Finally, we propose how EV-based treatment can be optimized to increase its immunomodulatory properties in an MI setting.

## 2. The Innate Immune System in Ischemic Injury and Repair of the Heart

The hostile environment of the infarcted area gives rise to necrotic and stressed cells that trigger sterile inflammation by exposing damage-associated molecular patterns (DAMPs). DAMPs include damaged or modified extracellular matrix components and intracellular constituents such as ATP, alarmins, or mitochondrial elements and have been extensively studied as therapeutic targets to prevent ischemic injury in the preclinical setting [26]. Pattern recognition receptors (PRRs) expressed by cardiac resident immune and non-immune cells recognize DAMPs, which induce a signaling cascade that leads to the release of pro-inflammatory cytokines and chemokines, such as TNFα, IL-1β, IL-6 and CC-chemokine ligand 2 (CCL2) [27,28]. These pro-inflammatory mediators, together with reactive oxygen species (ROS) present in the ischemic microenvironment, activate endothelial cells, causing increased expression of adhesion molecules on the endothelium [29]. Together, this culminates in the recruitment of neutrophils and monocytes from the bone marrow and the spleen into the ischemic region [30,31].

Neutrophils, monocytes, and macrophages are primarily responsible for the phagocytosis of cell debris [32,33]. In addition, they produce proteases that digest the tissue matrix, enabling the removal of dead material and allowing proper scar formation. The secretion of pro-inflammatory cytokines by these immune cells further stimulates cardiac inflammation [34]. While neutrophil numbers decline three days post-MI, monocytes continue to infiltrate in the ischemic area and differentiate into macrophages for several days [35,36].

**Figure 1:**
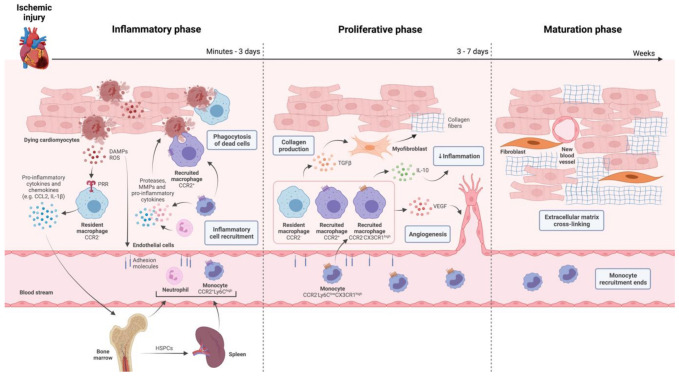
Role of innate immune cells on cardiac repair after myocardial infarction. Cell death caused by ischemic injury releases damage-associated molecular patterns (DAMPs) and reactive oxygen species (ROS), which initiates the inflammatory phase by being recognized by pattern recognition receptors (PRRs) on surrounding cells. Here, cardiac cells secrete pro-inflammatory cytokines and chemokines, leading to an intense influx of neutrophils and inflammatory monocytes into the cardiac ischemic area, responsible for phagocytosis and digestion of necrotic tissue. After a few days, this phase transitions into a proliferative phase where inflammation resolution occurs and a scar develops. Tissue macrophages, either resident or monocyte-derived (recruited), shift their polarization towards anti-inflammatory and produce transforming growth factor-β (TGFβ), interleukin-10 (IL-10) and vascular endothelial growth factor (VEGF), which leads in turn to collagen deposition by myofibroblasts, attenuation of inflammation and neovascularization, respectively. Within weeks, monocyte recruitment ends, and the scar matures via extracellular matrix cross-linking by fibroblasts. CCR2, CC-chemokine receptor 2. Ly6C, lymphocyte antigen 6C. MMPs, matrix metalloproteinases. CX3CR1, CX3C chemokine receptor 1.

Monocytes and macrophages follow a biphasic response to ischemic injury in the heart (Figure 1) [37]. In mice, circulating C-C chemokine receptor type 2 (CCR2)^+^, lymphocyte antigen 6C (LY6C)^high^ monocytes arrive at the injured region 30 min after MI thanks to the high concentration of CCL2 in the infarcted area. This pro-inflammatory monocytic population secretes IL-1β, IL-6, and TNFα, thereby contributing to continued cardiac inflammation and scavenge dead cells. Next to this initial wave of pro-inflammatory monocytes, there is a less intensive wave of pro-reparative CCR2-LY6C^low^, CX3C chemokine receptor 1(CX3CR1)^high^ monocytes due to the presence of chemokine fractalkine (CX3CL1) between day 5 and 16 after MI [38]. During this reparative phase, cardiac macrophages exhibit a more anti-inflammatory phenotype and produce IL-10 and transforming growth factor-β (TGFβ) as well as angiogenic factors, which promotes collagen production by fibroblasts and enhancement of the capillary density, respectively [39]. Although these two main subsets of circulating monocytes (LY6C^high^ and LY6C^low^) have been studied in mice [38], they are suggested to be equivalent to classical CD14^+^CD16^low^ and non-classical CD14^+^CD16^high^ monocytes in humans [40]. Clinical studies showed that high levels of classical monocytes in the blood of MI patients negatively affected their prognosis [40,41]. Nevertheless, the mechanism behind how monocyte subtypes contribute to cardiac injury and repair in human patients remains to be further elucidated [39].

Macrophages initiate the resolution of the inflammatory phase through the engulfment of damaged cells, which induces phenotypic changes in macrophages through the upregulation of the signaling pathways such as kinase AMPK [32,42]. On the other hand, neutrophils promote macrophage polarization into a more anti-inflammatory phenotype by releasing neutrophil gelatinase-associated lipocalin (NGAL) [43]. Therefore, macrophages are pivotal in the inflammatory response post-MI and its resolution, which makes them very interesting therapeutic targets. Moreover, macrophage depletion through liposomal clodronate treatment in the neonatal heart of mice prevented its regeneration from MI, strengthening this observation [44].

Left ventricular (LV) dysfunction can occur in case of dysregulation of this inflammatory response due to chronic low-grade inflammation, contributing to heart failure development [2]. A better understanding of this complex process will provide new therapeutic opportunities to protect the heart from immune-mediated damage. Accumulating evidence suggests that macrophages are responsible for orchestrating the therapeutic effect of cell therapy upon MI [24,45,46]. Meanwhile, the anti-inflammatory and immunomodulatory potential of EVs derived from different stem and progenitor cells is being investigated in cardiac injury and repair [47]. The following section will provide a comprehensive overview of the preclinical studies investigating the interaction between EVs secreted by stem and progenitor cells and monocytes/macrophages in the cardiac setting.

## 3. Extracellular Vesicles (EVs) and Their Interaction with Monocytes and Macrophages

EVs are endogenous carriers of biological material and can be classified into two major categories according to their origin in the cell: exosomes, also called small EVs (30–100 nm in diameter), and microvesicles (50–10,000 nm). Exosomes are generated within the endosomal compartment, whereas microvesicles are formed via the budding of the plasma membrane [48]. Given the complexity of distinguishing EVs based on their biogenesis, the term EVs will refer to small EVs in this review [49].

The therapeutic potential of EVs, derived mainly from stem and progenitor cells, has been explored in the context of myocardial repair after MI [50,51,52,53]. Embryonic stem cells (ESCs) and mesenchymal stem cells (MSCs) have been widely studied, however, ESCs have inherent ethical problems due to their origin, which MSCs can circumvent as they can be isolated from adult tissue upon informed consent [54]. Even though MSCs have a more limited potency to differentiate into different cell types compared to ESCs, their application in regenerative medicine has been successful in preclinical studies [55]. Cardiac progenitor cells (CPCs) have also been investigated in myocardial repair. These progenitor cells are derived from the heart, either fetal or adult, and can be isolated based on stem cell markers, such as Sca-1, or their clonogenic potential [56]. When CPCs are cultured under low-adhesion conditions, they form spherical aggregates, the so-called cardiosphere-derived cells (CDCs). CDCs are a mixture of stromal, mesenchymal, and progenitor cells and have distinct cell proliferation and maturation due to the cell-cell interactions [57].

A meta-analysis revealed that EVs derived from the previously mentioned cells showed an ability to reduce infarct size and improved the ejection fraction of the treated infarcted hearts in both small and large animal models [58]. The mechanism behind the cardioprotection provided by these vesicles remains not entirely understood. Still, EV treated groups often present an increase in angiogenesis and a reduction in cardiomyocyte apoptosis [51,52,53,59]. In addition, alleviation of fibrosis was also noticed in some studies [53,60]. More recently, the same EVs presented anti-inflammatory and immunomodulatory effects after being injected upon MI [47]. In general, there are two main mechanisms by which EVs released by ESCs, MSCs, CPCs, and CDCs achieve their immunomodulatory outcomes: reduction of monocyte infiltration in the ischemic cardiac tissue and modulation of macrophage polarization (Table 1). The following sections will address these mechanisms in more detail.

### 3.1. Reduction of Monocyte Infiltration

Preclinical research has shown the potential of targeting the CCR2-CCL2 signaling axis and, thus, pro-inflammatory monocyte infiltration in ischemic heart failure [61]. Likewise, EVs derived from MSCs, CPCs, CDCs, and endothelial cells, administrated either intravenously or directly in the heart, showed positive outcomes by reducing monocyte influx into the heart via different mechanisms.

The first study providing evidence that MSC-EVs can decrease cardiac inflammation was performed in a mouse model of myocardial ischemia/reperfusion injury, where the EVs were injected intravenously before reperfusion [51]. Here, a reduction of Ly6G^+^ neutrophils and MAC-3^+^ macrophages in the hearts was observed at day 1 and 3 post-MI as well as a decrease in circulating white blood cells at the same time points. Similar to MSC-EVs, CPC-EVs significantly decreased Ly6G^+^ neutrophils and Ly6C^high^ monocytes in the mouse cardiac tissue of a permanent ligation MI model when injected into the heart at both acute (2 days after ligation) and chronic phases (3 weeks after ligation) of ischemic heart failure [62]. There is limited evidence in these studies which mechanism of action was responsible for the observed immunomodulatory effects, but, perhaps, different mechanistic between them considering the different administration routes used.

The therapeutic role of EVs has been mostly attributed to the delivery of functional cargo to recipient cells. EVs can carry different biomolecules, including proteins and non-coding RNAs, in particular microRNAs (miRNAs) [63]. miRNAs are often analyzed as they can fine-tune cellular function by decreasing protein translation. Some of the following studies suggested that the transfer of different miRNAs via EVs can modulate leukocyte influx in the heart.

miR-24 has been described to limit vascular inflammation by regulating macrophage behavior in atherosclerosis and abdominal aortic aneurysm mouse model [64,65]. Considering this finding, it comes with no surprise that miR-24-3p was suggested to play a role in cardioprotection provided by EVs of different cells. For example, EVs secreted by endothelial cells overexpressing Krüppel-like factor 2, injected intravenously after reperfusion, were found to reduce Ly6C^high^ monocyte infiltration in the murine heart on day 3 post-MI [66]. Here, miR-24-3p was suggested to inhibit leukocyte recruitment from the bone marrow by downregulating CCR2 in these cells [66]. Similarly, MSC-EVs injected in the rat heart after permanent ligation significantly reduced CD68^+^ macrophages in the peri-infarct zone one week after MI and were enriched in miR-24-3p [67]. Collectively, these studies show the potential of miR-24-3p as an immunomodulator when administrated in the acute phase of MI.

Besides miR-24-3p, miR-146a-5p and miR-181b were also described to be enriched in EVs reducing the monocyte influx in the injured heart. Rats treated with CPC-EVs, administrated via the tail vein at different days after doxorubicin/trastuzumab-induced cardiac toxicity, demonstrated a reduction in the number of infiltrated monocytes one month after cardiac damage [68]. These EVs presented high numbers of miR-146a-5p, which was previously reported to inhibit pro-inflammatory cytokine secretion in human gingival fibroblasts [69]. Moreover, the target genes of miR-146a, including Traf6 and Irak-1, which are involved in the toll-like receptor (TLR) signaling pathway, were found to be downregulated in the EV treated cardiomyocytes. CDC-EVs injected in the heart of rats and pigs, 20–30 min after reperfusion, showed fewer CD68+ macrophages in the cardiac tissue of treated groups two days after MI [70]. miR-181b was enriched in CDC-EVs and has been depicted to regulate NF-κB signaling in endothelial cells, limiting vascular inflammation [71]. Thus, miR-146a-5p and miR-181b confer interesting anti-inflammatory properties to EVs.

Although most studies refer to miRNAs as being responsible for the therapeutic role of EVs, proteins have been reported to contribute as well. For instance, CPC-EVs administrated in the heart 1 h after ligation reduced CD68^+^ macrophages in the treated rats one month after EV injection [72]. Pregnancy-associated plasma protein-A (PAPP-A) was found to be responsible for the therapeutic effect. PAPP-A is a protease responsible for the cleavage of insulin growth factor-1 (IGF-1) binding protein-4, which transports IGF-1. Once released from its complex, IGF-1 can act as an immunomodulator in the heart [73]. IGF-1 factor is one of many growth factors that macrophages secrete to modulate their microenvironment. A study showed that mice overexpressing cardiac-specific IGF-1 led to a reduction in inflammatory Ly6C^high^ monocytes at day 3 and increased anti-inflammatory CD206^+^ macrophages at day 7 post-MI [74,75,76].

**Figure 2:**
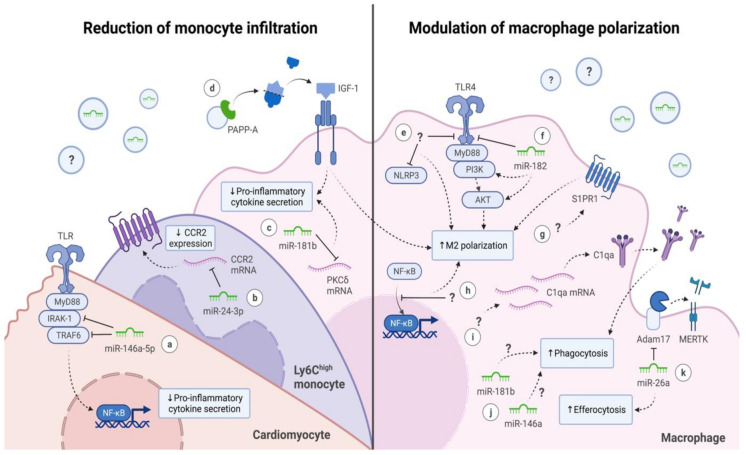
Mechanisms of action by which extracellular vesicles (EVs) exert their immunomodulatory effects after myocardial injury. EVs can modulate immune cell response after cardiac damage in two ways: reducing monocyte infiltration and modulating macrophage polarization. (**a**,**c**) Different microRNAs (miRNAs) are responsible for decreasing pro-inflammatory cytokine and chemokine secretion on different cell types in the heart, which reduces cardiac inflammation. (**b**) miR-24-3p decreases the expression of C-C chemokine receptor type 2 (CCR2) on the surface of monocytes, reducing their recruitment in the heart where high levels of C-C chemokine ligand 2 (CCL2) are present. (**d**) Pregnancy-associated plasma protein-A (PAPP-A) increases the extracellular levels of insulin growth factor-1 (IGF-1), which decreases the pro-inflammatory phenotype on macrophages. (**e**–**h**) Embryonic stem cell (ESC) and mesenchymal stem cell (MSC)-derived EVs stimulate anti-inflammatory M2 polarization on macrophages via different molecular mechanisms: inhibition of toll-like receptor 4 (TLR4) and NOD-, LRR- and pyrin domain-containing protein 3 (NLRP3) pathway, inhibition of nuclear translocation of nuclear factor-κB (NF-κB) or activation of phosphoinositide 3-kinase/protein kinase B (PI3K/AKT) and sphingosine-1-phosphate receptor 1 (S1PR1) pathway. (**i**,**j**) Cardiosphere-derived cell (CDC)-derived EVs increase phagocytosis activity by increasing complement factor C1qa expression or via unknown pathways. (**k**) CDC-EVs enriched in miR-26a suppressed disintegrin and metalloprotease 17 (Adam17) on macrophages, which sustains tyrosine-protein kinase Mer (MERTK) expression by decreasing the cleavage of this receptor, enhancing the efferocytosis ability of macrophages. A question mark is placed inside of an EV (circle) or before an arrow in case of the EV cargo responsible for the therapeutic effect is unknown. The mechanism of action exerted by the EVs is unknown when the question mark is positioned next to the arrow. Ly6C, lymphocyte antigen 6C.

In short, these studies indicate that EVs secreted from different progenitor and differentiated cells can inhibit the monocyte influx into the injured region via different mechanisms and, thus, are potentially attractive therapeutic agents to fight cardiac inflammation (Figure 2).

### 3.2. Modulation of Macrophage Polarization

Macrophages are the most abundant immune cells present in the heart after MI [77]. Macrophages exhibit high plasticity and adopt different polarization states depending on their microenvironment. Macrophage polarization in vivo is still not completely understood, however, M1 and M2 terminology is recognized as the extremes of this polarization spectrum [78]. In general, M1 macrophages secrete pro-inflammatory cytokines and are active in phagocytosis, whereas M2 macrophages promote tissue repair and are considered more anti-inflammatory [79,80]. Modulation of macrophage polarization has been emerging as an attractive therapeutic approach for inflammatory diseases. Stem- and progenitor-derived EVs were able to improve cardiac function in preclinical studies by modulating macrophage activity.

Interestingly, there is a difference in the capacity of stem- and progenitor-derived EVs to modulate macrophage polarization after MI as described in the literature so far (Table 1). In general, ESC- and MSC-EVs showed a capacity to polarize macrophages into an M2 phenotype, while CDC- and CPC-EVs seemed to modulate macrophage polarization into a phenotype with increased phagocytotic capacity. Differences between the donor cells from which these EVs arise may explain this, however, the cause behind the different macrophage polarization awaits further investigation.

ESC-EVs induced M2 macrophage polarization by decreasing nitric oxide synthase (iNOS) expression and increasing CD206 levels in the cardiac tissue of a doxorubicin-induced cardiomyopathy mouse model two weeks after multiple intraperitoneal injections on different days [81]. In addition, the heart of ESC-EV treated animals showed a decrease of pro-inflammatory cytokines TNFα and IL-1β expression) while increasing the anti-inflammatory cytokine IL-10 [81]. Equally important, these EVs were associated with reduced inflammasome markers, such as NLRP3, which are activated in response to the release of DAMPs by stressed or dying cells consequential to MI [82]. The anti-inflammatory effect of ESC-EVs was mainly attributed to inhibition of the TLR adaptor protein MyD88 and consequent non-activation of the MAPK signaling pathway via decreased phosphorylation of P38 and JNK [81,83].

EVs secreted by bone marrow and adipose tissue-derived MSCs showed an anti-inflammatory effect in vivo in MI and dilated cardiomyopathy models. Groups treated with MSC-EVs shown a decrease in pro-inflammatory cytokines, such as IL-1β, IL-6, and TNFα, which contrasted with a rise of anti-inflammatory cytokines, namely IL-10 [84,85,86,87]. The gene expression and protein levels of these cytokines were measured in the treated tissue and the serum of treated animals, respectively. In general, EVs secreted by MSCs promoted M2 polarization by increasing Arginase-1 (Arg1) and CD206 expression and decreasing the manifestation of M1 markers, such as iNOS, CD86, CD11b, and CD11c. This was observed both in the cardiac tissue of mouse and rat, intramyocardially and intravenously injected, and during in vitro culture of macrophages in the presence of EVs [84,85,86,87]. For in vitro studies, macrophages were treated with MSC-EVs before exposure to hypoxia or lipopolysaccharide (LPS) or after treatment with LPS [85,86,87]. Altogether, these data show the ability of MSC-EVs to modulate macrophage polarization towards the M2 phenotype.

Human CDC-EVs, injected intramyocardially after reperfusion, modulated macrophage polarization in treated rats and pigs [70]. Here, cardiac macrophages had lower levels of iNOS, Arg1, and TNFα whereas IL-1β was increased in treated animals. This gene expression does not fit into the M1 or M2 classification, however, naïve bone marrow-derived macrophages treated with CDC-EVs in vitro showed higher phagocytic activity compared to M1 polarization control. In another study, murine naïve bone marrow-derived macrophages presented increased gene expression of iNOS, Arg1, TNFα and IL-1β as well as enhanced phagocytosis when stimulated with CDC-EVs [88]. Later, CDC-EVs demonstrated a capacity to enhance macrophage efferocytosis in rodents, thereby promoting the uptake of apoptotic cells and, thus, inflammation resolution [89]. In addition, Y RNA fragment, a small non-coding RNA present in human CDC-EVs, increased the secretion of IL-10 in bone marrow-derived macrophages in vitro [90]. Pigs administrated intrapericardially with CDC-EVs presented an increase of circulating M2 monocytes (CD14+, CD163+) in the peripheral blood 24 h after treatment [91]. Arg1 was also increased in the pericardial fluids of the treated group. The potential of EVs released by human-induced pluripotent stem cell-derived CPCs was investigated in human monocyte-derived macrophages in vitro [62]. These EVs decreased M1 markers, such as CD80 and CD86, and elevated M2 markers, namely CD206 and CD163, in naïve macrophages. Collectively, CDC-EVs seem to regulate several genes attributed to M1 and M2 polarization, and stimulate phagocytic activity in macrophages.

MSC- and CDC-EVs modulated macrophage polarization in a dose-dependent manner in vitro [70,85,88]. Of note are some studies that used pre-treated cells to produce EVs. MSCs submitted to hypoxic conditions (1% oxygen) for 48 h [92] or treated with lipopolysaccharide (LPS) for 24 h [85] secreted EVs with stronger anti-inflammatory effects.

It appears that the functional transfer of multiple miRNAs is involved in the mechanism of action by which EVs modulated macrophage polarization (Figure 2). MiR-182 has been previously described as a mediator of macrophage polarization via toll-like receptor 4 (TLR4) [93]. MSC-EV treatment, and miR-182 transfection, inhibited TLR4/NF-κB and activated PI3K/AKT signaling pathway, which promoted M2 polarization [87]. MiR-181b enriched in CDC-EVs downregulated protein kinase C δ (PKCδ) in macrophages, which is responsible for inflammatory gene expression [70]. PKCδ inhibitors are known to promote cardioprotection in the human myocardium [94]. EVs released by CDCs were also reported to transport high levels of miR-26a [89]. The delivery of this miRNA via EVs enhanced efferocytosis activity in the target cells by suppressing Adam17, which in turn sustains the expression of MerTK. MerTK has been linked with acute inflammation resolution by promoting efferocytosis in macrophages [32]. In addition, CDC-EVs induced complement factor C1qa expression, a phagocytosis facilitator, contributing to increased phagocytosis activity in target macrophages [89]. In brief, stem and progenitor cell-derived EVs seem to interact with macrophages, modulating their polarization into anti-inflammatory or increasing their efferocytosis capacity. Overall, EV treated groups presented lower pro-inflammatory cytokines in circulation and in the heart, thereby decreasing cardiac inflammation and oxidative stress. 

## 4. Improving EV-Based Therapies for Immunomodulation in MI Treatment

EVs hold several advantages that make them an exciting therapeutic alternative over cell therapy due to their intrinsic properties. For example, they can survive in the extracellular space, bypass biological barriers and deliver active biological cargo to recipient cells [95]. Nevertheless, multiple advancements should be tackled in the EV field for their future clinical application in the post-MI setting [22]. Firstly, there is need for more standardized EV-production and isolation methods at industrial-scale to guarantee the reproducibility and GMP quality of therapeutic EVs [96]. Secondly, the optimal storage conditions of EVs remain to be further evaluated. Thirdly, future research should determine the best administration route, time window (before or after reperfusion), and the number of infusions needed for a sustained effect. Injecting EVs systemically offers the advantage of targeting cells in the bone marrow more easily while administrating them locally in the heart makes them more likely to target cardiac macrophages and other cardiac cell types. We refer to the review of Kennedy et al. for a more comprehensive overview of the current limitations of EV research before moving to clinical translation [97].

In the future, bioengineering of EVs may be used to improve the therapeutic properties of EVs [63]. For instance, by modifying EVs to increase the loading of therapeutic cargo, or by equipping them with molecules that enhance their delivery to target cells. In the following sections, we will discuss how EVs can be modified to improve their therapeutic potential.

### 4.1. Modifying EV Cargo

EVs can transmit information to recipient cells without delivering their content but by acting at the cell surface of the targeted cell [98]. For instance, EVs derived from dendritic cells activate T lymphocytes via the major histocompatibility complex-peptide complexes displayed at the surface of EVs [99]. However, the main feature of EVs is to enclose and transmit bioactive molecules by a lipid bilayer. For this to happen, EVs need to be internalized by recipient cells and either be directed to the lysosome, where they are degraded and their content recycled, or release their intraluminal content directly into the cytoplasm [21]. Thus, EVs need to be internalized to deliver their cargo to acceptor cells.

EVs comprise various proteins as well as coding and non-coding RNAs as cargoes in their lumen [100]. The abundance and type of EV cargo are cell-specific and can be influenced by the physiological and pathological state of donor cells [21]. Additionally, EVs are not a homogenous population, but rather heterogeneous subpopulations with different proteomic and nucleic acid in their composition, mediating different responses in recipient cells [101]. Overall, this emphasizes the challenge of studying EVs as therapy because they are not identical between them and their composition varies according to donor cells and their physiological state. Consequently, efforts must be made to characterize EV content in-depth, allowing us to identify which cargo is responsible for reducing cardiac inflammation in vivo to fine-tune EV content before clinical translation.

Most research focused on EV-derived miRNAs when considering the therapeutic potential of EV cargo, however, it is speculated that one would need, on average, over 100 EVs to detect a single copy of an abundant miRNA [102]. Bearing in mind the limited availability of a specific miRNA present in EVs, studying the protein content of EVs offers an opportunity to unravel the mechanism behind the therapeutic role of EVs [103]. The application of multiple omics will allow in-depth investigation of the therapeutic content of EVs in the near future. The bioactivity of EVs can be improved in this way by enriching them with specific bioactive molecules via their overexpression in the EV-secreting cells or transfection of the EVs [63]. These biomolecules can be naturally expressed by the donor cells or be exogenous.

The chemokine CCL2-CCR2 signaling axis has been investigated over the last years as a potential target to decrease monocyte recruitment [104,105]. Inflammatory monocytes depend on the chemokine receptor CCR2 to travel to the injured tissue, which makes this receptor an interesting therapeutic target. Gene therapy using CCR2-silencing short interfering RNA (siRNA) encapsulated in lipid nanoparticles prevented monocyte infiltration in the cardiac tissue, attenuating infarct inflammation and limiting LV remodeling [105]. EVs could also be explored to either silence or downregulate the expression of this receptor by carrying siRNA or miRNA, respectively, adding therapeutic value to their intrinsic properties. KLF2-overexpressing endothelial cells released EVs enriched in miR-24-3p that seems to be able to downregulate CCR2 [66]. Nevertheless, undesired consequences can arise from depleting monocytes post-MI, such as prolonged cardiac inflammation due to insufficient clearance of cardiac dead tissue and reduction of macrophages necessary for cardiac repair [106].

Alternatively, cardiac macrophages, either tissue-resident or monocyte-derived, could be pushed toward an anti-inflammatory phenotype by manipulating their gene expression [107]. Nuclear receptor subfamily 4 group A member 1 (NR4A1) and interferon regulatory factor 5 (IRF5) are within the transcription factors that showed promising results in this regard [108,109]. Some of the preclinical studies included in this review identified EVs that can favor M2 polarization in macrophages, yet, it is not completely clear which mechanism the EVs can modulate macrophage phenotype. On the other hand, EVs could be investigated as drug delivery systems to target a specific subset of macrophages and carry the right macromolecular content to stimulate this phenotype. However, the current knowledge about how macrophages select reparative fate over inflammatory in humans is still limited [110]. Future research concerning the molecular mechanism by which monocytes and macrophages acquire anti-inflammatory phenotype will be fundamental to develop EV therapies to support this phenomenon.

### 4.2. Enhancing Cell-Specific Targeting

An EV must bind to its recipient cells to exert its biological function. It is known that EVs are capable of binding to specific target cells and that protein and lipid composition of their surface influence their targeting behavior. For instance, CD47 has been reported to inhibit EV uptake by macrophages [111] while phosphatidylserine, an “eat-me” signal usually found on apoptotic cells, is recognized by macrophages, which led to EV internalization [112]. Additionally, EVs released by cardiomyocytes during homeostasis were found to express phosphatidylserine on their surface, which was recognized by phagocytic receptors in cardiac resident macrophages [113]. Together, this evidence shows phosphatidylserine as a promising candidate for targeting macrophages by EVs.

Surface engineering of EVs offers an exciting opportunity to optimize EV performance in vivo [114]. There are several strategies to modify the moieties of EVs to enhance their cell targeting properties. An interesting approach is to modify EVs with a recombinant fusion protein/nanobody complex, which binds to a specific EV moiety on its surface and a selective receptor present on the cell of interest [115]. In addition, there is the option of linking EVs with diverse macromolecules, such as antibody fragments, by fusing “scaffold” proteins present in EVs with the molecules of interest [116]. This strategy can be further applied to modify not only EV surface and, thus, increase EV tropism, but also its cargo inside the vesicle lumen, which enhances their therapeutic value.

Membrane fusion has also been used in nanomedicine to obtain biosynthetic hybrid vectors. This approach can also be applied to EVs by fusing them with, for example, functionalized liposomes, improving their cellular delivery [117]. Likewise, there is the option of combining EVs derived from different parent cells as well. A study applied this method to fuse MSC-EVs with the membrane of monocyte-derived EVs to mimic the recruitment feature of monocytes following MI. This resulted in enhanced targeting to the injured myocardium and improved cardiac function [118]. Another advantage of this method is the opportunity to deliver EVs via intravenous route, which is more convenient than intramyocardial injection.

Another essential point is to choose a selective feature of the cell of interest when considering EV targeting optimization. Nevertheless, the characterization of monocyte and macrophage populations in cardiac ischemic is still in its infancy. Single-cell transcriptomics analysis will improve our understanding of which monocyte and macrophage population should be targeted and when [106]. This advance will improve EV tropism as well as the timing and the route of EV injection in the patient.

It is generally accepted that EVs need to be internalized to exert their function on the recipient cells, however, it is unclear whether this is a requisite in monocytes and macrophages. This process is likely to depend on the mechanism of action by which EVs interact with recipient cells and whether it is due to the biological cargo or a moiety on their surface. If EV uptake is required, strategies to increase their internalization and endolysosomal escape, such as EV modification with cationic lipids, should be considered [119]. Nevertheless, the mechanisms by which EV cargo is delivered and how EVs avoid its degradation are still poorly understood [95].

## 5. Conclusions

It has become evident over the last decade that monocytes and macrophages play a central role in cardiac repair following MI, which makes them attractive therapeutic targets. EVs have been investigated due to their regenerative properties in cardiovascular settings. Here, the described research collectively shows that immunomodulation provided by stem and progenitor cell-derived EVs can be one of the mechanisms of action by which EVs offer cardioprotection post-MI. Further research addressing the challenges in the EV field will be key to fine-tune EV therapy in the future. These include characterization of therapeutic content of EVs and improvement of its cell targeting as well as what is the best administration route and its time window. Although there is still a long way to pursue before translation into clinics is possible, there is an exciting avenue for immunomodulatory therapy in MI provided by EVs.

## Figures and Tables

**Table 1:** Immunomodulation by extracellular vesicles in myocardial infarction and cardiotoxicity models.

EV Source	Experimental Model	EV Administration	EV Isolation Method	Functional EV Content	Molecular Mechanism	Biological Effect	REF
*Reduction of monocyte infiltration*							
Human ESC-derived MSCs	MI mouse model (I/R)	Intravenous; 5 min before reperfusion	SEC	Unknown	Unknown	Reduced neutrophil and macrophage infiltration in the hearts and WBC count.	[51]
Rat bone marrow MSCs	MI rat model (PL)	Intramyocardial (2 different sites); immediately after ligation	Precipitation	miR-24-3p	Unknown	Decreased of CD68+ macrophages in the peri-infarct zone.	[67]
Human CDCs	MI rat and pig model (I/R)	Intramyocardial (10 sequential points in pig); 20 min after reperfusion in rat/30 min after reperfusion in pig	Ultrafiltration and PEG precipitation	miR-181b	Downregulation of protein kinase C δ	Reduced of CD68+ macrophages within infarcted tissue and increased phagocytosis capacity of macrophages.	[70]
Human CPCs	MI rat model (PL)	Intramyocardial (3 different sites); 60 min after ligation	UC	PAPP-A	Unknown	Decreased CD68+ macrophages within infarcted tissue.	[72]
Human iPSC-derived CPCs	MI mouse model (PL)	Transcutaneous (three peri-infarcted areas); 2 days (acute) or 3 weeks (chronic) after PL	UC	Unknown	Unknown	Decreased Ly6Chigh monocytes in the heart and levels of pro-inflammatory cytokines.	[62]
Human CPCs	Dox/Trz-induced cardiotoxicity rat model	Intravenous; Days 5, 11, and 19	UC	miR-146a-5p	Inhibition of Traf6 and Irak1	Reduced CD68+ macrophages infiltrates in the heart.	[68]
Human and mouse KLF2-overexpressing endothelial cells	MI mouse model (I/R)	Intravenous; immediately after reperfusion	UC	miR-24-3p	CCL2/CCR2 axis	Inhibited Ly6Chigh monocytes recruitment from bone marrow by inhibiting CCR2 expression.	[66]
*Modulation of macrophage polarization*							
Mouse ESCs	Dox-induced cardiotoxicity mouse model	Intraperitoneal (3 injections in 3 different days between Dox treatment)	Precipitation (Exoquick TC)	Unknown	Inhibition of MyD88 /P38/JNK and NLRP3 pathway	Increased M2 macrophages and anti-inflammatory cytokine IL-10.	[81]
Mouse bone marrow MSCs	MI mouse model (I/R)	Intramyocardial (3 different sites); immediately after reperfusion	UC	miR-182	Inhibition of TLR4/NF-κB pathway and activation of PI3K/AKT pathway	Promoted M2 polarization in macrophages.	[87]
Rat adipose tissue MSCs	MI rat model (PL)	Intravenous; 60 min after ligation	UC	Unknown	Activation of S1P/SK1/S1PR1 signaling	Promoted M2 polarization in macrophages.	[86]
Rat bone marrow MSCs	MI mouse model (PL)	Intramyocardial (4 different sites); immediately after ligation	Density-gradient UC	Unknown	Inhibition of nuclear translocation of NF-κB p65 and activation of phosphorylation of AKT1 and AKT2	Decreased the production of pro-inflammatory cytokines and increased M2 polarization in macrophages.	[85]
Mouse bone marrow MSCs	Dox-induced dilated cardiomyopathy mouse model	Intravenous; 7 days after Dox treatment	UC	Unknown	Activation JAK2-STAT6 pathway	Decreased circulating pro-inflammatory cytokines and M1 macrophages in the heart, while increasing M2 macrophages.	[84]
Human CDCs	MI rat model (I/R)	Intramyocardial; 10 min after reperfusion	Ultrafiltration	Y RNA fragment	Unknown	Increased IL-10 secretion in macrophages.	[90]
Human CDCs	MI rat and mouse model (I/R)	Intramyocardial (3 different sites); 20 min after reperfusion	Ultrafiltration	miR-26a	Suppression of Adam17 and upregulation of C1qa	Inducted of C1qa and MerTK expression in macrophages, which enhances phagocytosis and efferocytosis.	[89]
Pig CDCs	MI pig model (I/R)	Intrapericardially; 3 days after MI	Ultrafiltration	Unknown	Unknown	Increased circulation of M2 monocytes.	[91]
Human CDCs	In vitro	NA	Precipitation (ExoQuick-TC) or ultrafiltration	miR-146a	Unknown	Increased phagocytosis in macrophages.	[88]

Abbreviations: ESC, embryonic stem cell; MSC, mesenchymal stem cell; CDC, cardiosphere-derived cell; CPC, cardiac progenitor cell; KLF2, Krüppel-like factor 2; MI, myocardial infarction; I/R, ischemia/reperfusion; PL, permanent ligation; Dox, doxorubicin; Trz, trastuzumab; NA, not applicable; SEC, size exclusion chromatography; UC, ultracentrifugation; PAPP-A, pregnancy-associated plasma protein-A; CCL2, C-C motif chemokine ligand 2; CCR2, C-C chemokine receptor type 2.

## Data Availability

Not applicable.
